# Cooperation of ETV6/RUNX1 and BCL2 enhances immunoglobulin production and accelerates glomerulonephritis in transgenic mice

**DOI:** 10.18632/oncotarget.7687

**Published:** 2016-02-23

**Authors:** Eva Bauer, Michaela Schlederer, Ruth Scheicher, Jaqueline Horvath, Petra Aigner, Ana-Iris Schiefer, Renate Kain, Heinz Regele, Gregor Hoermann, Günter Steiner, Lukas Kenner, Veronika Sexl, Andreas Villunger, Richard Moriggl, Dagmar Stoiber

**Affiliations:** ^1^ Ludwig Boltzmann Institute for Cancer Research, Vienna, Austria; ^2^ Clinical Institute of Pathology, Medical University of Vienna, Vienna, Austria; ^3^ Institute of Pharmacology and Toxicology, University of Veterinary Medicine, Vienna, Austria; ^4^ Institute of Pharmacology, Center for Physiology and Pharmacology, Medical University of Vienna, Vienna, Austria; ^5^ Department of Laboratory Medicine, Medical University of Vienna, Vienna, Austria; ^6^ Cluster Arthritis and Rehabilitation, Ludwig Boltzmann Society, Vienna, Austria; ^7^ Division of Rheumatology, Department of Internal Medicine III, Medical University of Vienna, Vienna, Austria; ^8^ Unit of Pathology of Laboratory Animals, University of Veterinary Medicine, Vienna, Austria; ^9^ Division of Developmental Immunology, Biocenter, Medical University Innsbruck, Innsbruck, Austria; ^10^ Tyrolean Cancer Research Institute, Innsbruck, Austria; ^11^ Institute of Animal Breeding and Genetics, University of Veterinary Medicine, Vienna, Austria

**Keywords:** ETV6/RUNX1, BCL2, glomerulonephritis, lymphoma, autoimmunity, Immunology and Microbiology Section, Immune response, Immunity

## Abstract

The t(12;21) translocation generating the *ETV6/RUNX1* fusion gene represents the most frequent chromosomal rearrangement in childhood leukemia. Presence of ETV6/RUNX1 alone is usually not sufficient for leukemia onset, and additional genetic alterations have to occur in ETV6/RUNX1-positive cells to cause transformation. We have previously generated an *ETV6/RUNX1* transgenic mouse model where the expression of the fusion gene is restricted to CD19-positive B cells. Since BCL2 family members have been proposed to play a role in leukemogenesis, we investigated combined effects of ETV6/RUNX1 with exogenous expression of the antiapoptotic protein BCL2 by crossing *ETV6/RUNX1* transgenic animals with *Vav-BCL2* transgenic mice. Strikingly, co-expression of ETV6/RUNX1 and BCL2 resulted in significantly shorter disease latency in mice, indicating oncogene cooperativity. This was associated with faster development of follicular B cell lymphoma and exacerbated immune complex glomerulonephritis. *ETV6/RUNX1-BCL2* double transgenic animals displayed increased B cell numbers and immunoglobulin titers compared to *Vav-BCL2* transgenic mice. This led to pronounced deposition of immune complexes in glomeruli followed by accelerated development of immune complex glomerulonephritis. Thus, our study reveals a previously unrecognized synergism between ETV6/RUNX1 and BCL2 impacting on malignant disease and autoimmunity.

## INTRODUCTION

The t(12;21)(p13;q22) chromosomal translocation gives rise to the *ETV6/RUNX1* (*TEL/AML1*) fusion gene. *ETV6/RUNX1* represents the most abundant translocation product in pediatric cancers with an incidence of up to 25% in children with B lymphoblastic leukemia (B-ALL) [1, 2]. Although this type of leukemia exhibits no high-risk features and responds well to therapy, relapses occur in about 20% of cases [3–6].

ETV6/RUNX1 has been shown to lead to an arrest in B cell development at the transition from pro- to pre-B cells and this is paralleled by expansion of pro-B cells [7]. The *ETV6/RUNX1* fusion was found to occur *in utero* in fetal hematopoiesis [8, 9], but disease outbreak is usually not detected in children before the age of two years. ETV6/RUNX1 positive B-ALL is diagnosed during childhood, with a peak incidence between three and six years of age [10]. This suggests that the translocation product alone is not sufficient for leukemia onset [11–13]. Indeed, previous reports have shown that *ETV6/RUNX1* is a weak oncogene and requires secondary mutations for manifestation of the disease [11, 14, 15]. We have previously generated a mouse model for ETV6/RUNX1 where transgene expression is driven by the *Cd19* promoter [16]. Thus, *ETV6/RUNX1* expression is restricted to B cells after *Pax5* is switched on during B cell development. In line with other animal models [14, 15, 17–20] we failed to detect leukemia in our transgenic mice, but we observed abnormal B cell maturation associated with increased ROS levels in the B cell compartment as well as increased frequency of pre- and immature B cells [16].

Members of the B cell lymphoma 2 (BCL2) protein family are crucial regulators of cell survival. Transgenic BCL2 overexpression promoted the development of B cell cancers, demonstrating its oncogenic potential (reviewed in [21]). BCL2 family member deregulation can also mediate chemotherapeutic or targeted drug resistance (reviewed in [22]). Furthermore, BCL2 family proteins are critically involved in autoimmune processes [23–27]. Several members of the BCL2 family play critical roles in leukemia development. Loss of BCL2 modifying factor (*BMF)*, a proapoptotic member of the BCL2 family, was recently shown in a single nucleotide polymorphism (SNP) array study to play a role in the development and relapse of ETV6/RUNX1 positive leukemia [28]. Interestingly, patients with *ETV6/RUNX1*-positive ALL exhibited a unique expression pattern of 16 key apoptosis genes, including BCL2 family members [29]. BCL2 overexpression has also been reported to act as a driver in follicular lymphomagenesis and as a cooperating second hit in humans or mouse models of Burkitt lymphoma [30–34]. However, the biological function of members of the BCL2 family in ETV6/RUNX1^+^B cells remains largely unexplored.

We hypothesized that ETV6/RUNX1^+^ B cells might benefit from overexpression of antiapoptotic BCL2 and that this should facilitate tumor outgrowth. To test this, we crossed the B cell-specific *ETV6/RUNX1* transgenic (E/R^tg^) mice [16] to *Vav-BCL2* transgenic mice [35]. The latter mouse strain is predisposed to develop follicular lymphoma with age and can develop a kidney disease, namely glomerulonephritis of an autoimmune type [36]. Here, we show that combined expression of *ETV6/RUNX1* and *BCL2* leads to significantly shorter disease latency in mice. Importantly, the ETV6/RUNX1 fusion product and the antiapoptotic protein BCL2 cooperate in the development and progression of follicular lymphoma. In addition, autoimmune glomerulonephritis was significantly more aggravated than in *Vav-BCL2*^tg^ mice. Double transgenic animals displayed increased B cell levels and autoreactive immunoglobulin (Ig) production compared to *Vav-BCL2*^tg^ mice. This led to deposition of immune complexes in glomeruli, resulting in glomerulonephritis. Our study therefore demonstrates a novel cooperative activity of ETV6/RUNX1 and BCL2 for glomerulonephritis and lymphoma development.

## RESULTS

### Mice harboring *ETV6/RUNX1* and *BCL2* transgenes display significantly decreased survival

We hypothesized that overexpression of BCL2 in context with ETV6/RUNX1 should accelerate B cell transformation. Thus, we combined E/R^tg^ mice [16] with one of the most frequent driver mutations in B cell neoplasias, namely the BCL2 oncoprotein. E/R^tg^ mice were bred with *Vav*-*BCL2* transgenic mice [35] to assess whether the antiapoptotic protein BCL2 would cooperate with ETV6/RUNX1 to initiate leukemia. Four groups of mice were obtained: double transgenics carrying both transgenes (E/R^tg^;BCL2^tg^), single transgenic mice that harbor either the *ETV6/RUNX1* (E/R^tg^) or the *BCL2* transgene (BCL2^tg^) and mice without any transgene, serving as wildtype controls. All mice were born at normal Mendelian ratio, were viable and showed no abnormalities at birth.

**Figure 1:**
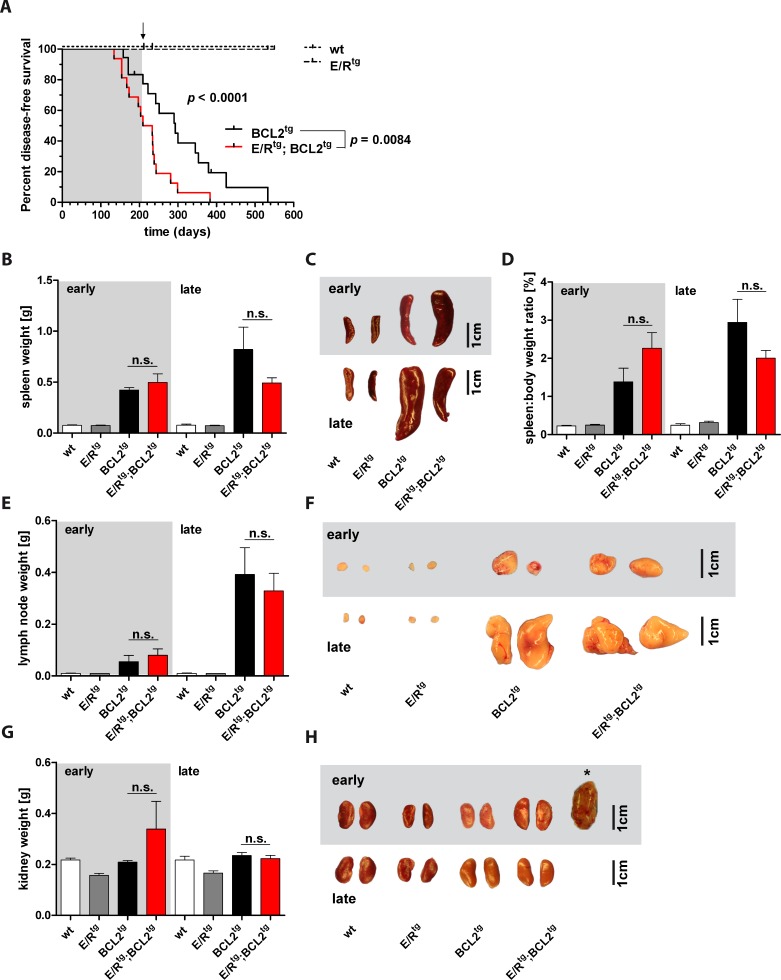
E/R^tg^;BCL2^tg^ mice display shortened disease latency **A** Kaplan-Meier plot showing disease-free survival of the four mouse groups. E/R^tg^ and wildtype (wt) mice stayed healthy over the indicated time period. E/R^tg^;BCL2^tg^ mice succumbed to disease significantly earlier as calculated by Log-rank (Mantel-Cox) test for all four groups and Chi square test for the two groups that develop disease. *n* = 16-18 mice per group, *p* values are considered as follows: *p < 0.05, **p < 0.01, and ***p < 0.001. Mice were divided into an early and a late disease group at 50% survival of the most affected (double transgenic) group (corresponding to 30 weeks of age), as indicated by the arrow and further on by the background color (grey indicates early, white indicates late disease). **B**, **D**, **E**, **G** Statistical analysis of spleen weight (B), spleen-to-body weight ratio (D), lymph node (the two largest abdominal lymph nodes of each mouse were used for analysis) (E) and kidney (G) weight (2 kidneys per mouse) are depicted. We failed to detect significant differences between double transgenic and BCL2^tg^ mice as examined by one-way analysis of variance (ANOVA) with Tukey's multiple comparison post-test, *n* ≥ 3 mice per group, means ± SEM are shown. *p* values are indicated only for the diseased groups (BCL2^tg^ *vs*. E/R^tg^;BCL2^tg^). **C**, **F**, **H** Representative pictures of spleens (C), lymph nodes (F) and kidneys (H) of early and late diseased mice and their corresponding controls are displayed. Asterisk in (H) shows the case of a massively enlarged kidney observed in an early diseased double transgenic mouse. Scale bar, 1 cm.

We observed that E/R^tg^;BCL2^tg^ mice exhibited a significantly shorter lifespan compared to BCL2^tg^ mice (221 *vs*. 293 days median survival) (Figure 1A). E/R^tg^ and wildtype mice remained healthy over an examination period of 80 weeks (550 days). The phenotype of diseased mice was heterogeneous depending on disease latency. Mice that succumbed to their disease at an early time point showed different symptoms (or gross pathology in organs) than animals that diseased at a late time point. We therefore decided to introduce an operational cutoff to divide the cohort into an early and a late disease group at the time point of 50% survival of the most affected, i.e. the double transgenic group at 30 weeks of age.

White blood cell counts were initially elevated in E/R^tg^;BCL2^tg^ and BCL2^tg^ mice [36] compared to E/R^tg^ and wildtype mice but started to decline between 12 and 18 weeks of age (Figure S1A, B). Red blood cell counts were comparable in all four groups but also decreased upon disease progression. Platelets were reduced in E/R^tg^;BCL2^tg^ and BCL2^tg^ mice when compared to E/R^tg^ and wildtype mice and further decreased in diseased mice (Figure S1A).

Diseased mice exhibited splenomegaly (Figure 1B, 1C) and enhanced spleen-to-body weight ratio (Figure 1D) up to 30 weeks of age. Increased spleen weight as well as spleen-to-body weight ratio in early diseased mice was more pronounced in double transgenic compared to BCL2^tg^ animals while in the late disease group this effect was inverted. The vast majority of BCL2^tg^ and all double transgenic mice displayed enlarged lymph nodes at the time point of analysis. Abdominal lymph nodes of double transgenic mice were larger when compared to BCL2^tg^ mice in early disease while this was inverted in the late disease phase (Figure 1E, 1F).

Despite all these signs of neoplastic hematopoiesis, we failed to detect overt leukemia in any of the transgenic mice except for one mouse in the double transgenic cohort (Figure S2A-C). This single mouse exhibited a strongly elevated white blood cell count when analyzed at 42 weeks of age (Figure S2A, B) with immunophenotyping confirming expansion of myeloid cells (CD11b^+^) (Figure S2C).

Of note, analysis of the liver, frequently infiltrated by BCL2-transgenic lymphocytes, did not reveal significant differences in weight and size between diseased and healthy cohorts (Figure S1C, D). Most significantly, E/R^tg^;BCL2^tg^ and BCL2^tg^ mice displayed enlarged kidneys with anemic appearance. While kidneys of double transgenic mice were clearly more affected in both early and late disease phases, elevated kidney weight was only observed in the early disease group (Figure 1G, 1H). This suggests that the lymphoma phenotype was either more dominant at late disease stages or that mice with severe kidney disease died earlier than unaffected animals.

### High lymphocyte infiltration in E/R^tg^;BCL2^tg^ mice

**Figure 2:**
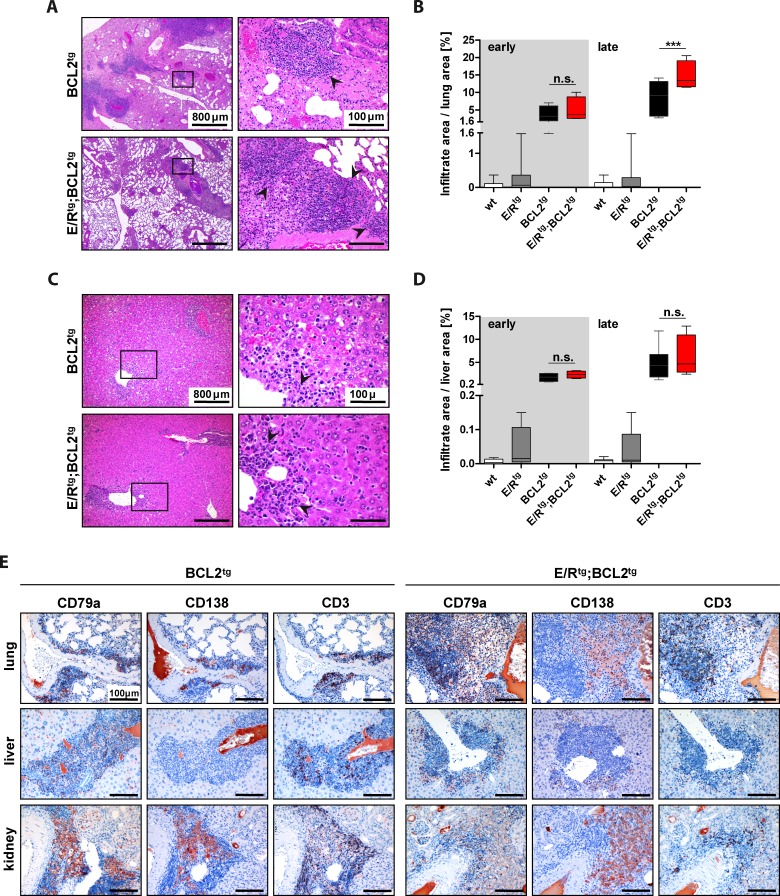
Lungs and livers of BCL2^tg^ and E/R^tg^;BCL2^tg^ mice harbor infiltrates composed of B, T and plasma cells **A** and **C.** Representative HE stained sections of lungs (A) and livers (C) of late disease BCL2^tg^ and double transgenic animals are shown. Infiltrates (arrowheads) are found alongside vessels. Scale bars as indicated in the picture. **B**, **D** Quantification of infiltrate area per lung area (B) and liver area (D), respectively, *n* ≥ 3 animals per group and at least 2 consecutive sections per mouse; data are presented as box plots, whiskers indicate min and max. Statistical analysis was examined by one-way ANOVA with Tukey's multiple comparison post-test, *p* values are considered as follows: *p < 0.05, **p < 0.01, and ***p < 0.001. *p* values are indicated only for the diseased groups (BCL2^tg^ *vs*. E/R^tg^;BCL2^tg^). **E** Sections of infiltrated lung, liver and kidney of BCL2^tg^ and double transgenic mice stained for CD79a (B cells), CD138 (plasma cells) and CD3 (T cells), respectively. Scale bars, 100 μm.

**Figure 3:**
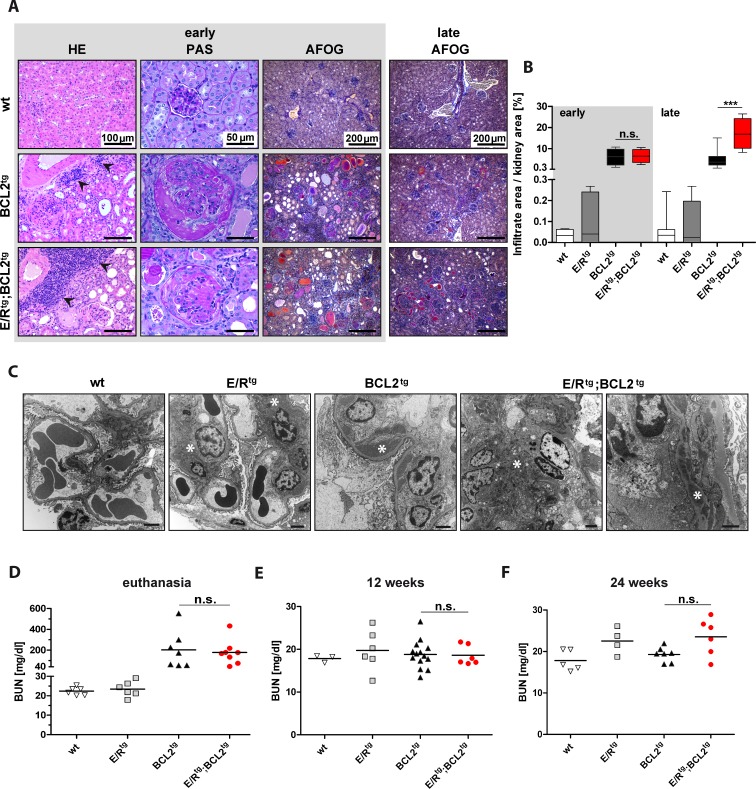
Double transgenic mice and BCL2^tg^ mice display glomerulonephritis and protein deposits in kidneys **A** Representative pictures of kidney sections of wt, BCL2^tg^ and E/R^tg^;BCL2^tg^ mice stained with HE (first column), showing infiltrates in kidneys (indicated by arrowheads). Periodic acid Schiff (PAS) staining indicates enlarged glomeruli and crescents in double transgenic and BCL2^tg^ mice (second column). Third and fourth columns show acid fuchsin orange G (AFOG) staining of kidney sections from early disease and late disease animals, respectively. Protein deposits are displayed in orange, pink and red. Scale bars as indicated in the pictures. **B** Quantification of infiltrate area per kidney area, *n* ≥ 3 animals per group and at least 2 consecutive sections per mouse, box plots with whiskers indicating min and max are shown. **C** Representative transmission electron microscopy pictures of all mouse groups. Wildtype glomerulum shows no pathological abnormalities. Scale bar, 2 μm. For E/R^tg^ a glomerulum with open capillaries and without abnormality except for mesangial matrix increase and mesangial electron dense deposits but no hypercellularity is shown. Scale bar, 2 μm. BCL2^tg^ shows a capillary loop with subendothelial electron dense deposits and basement membrane duplication. Scale bar, 2 μm. The most severe phenotype was seen in E/R^tg^;BCL2^tg^ mice indicated by mesangial matrix and cell increase with mesangial as well as subendothelial electron dense deposits, basement membrane thinning, endothelial cell activation and hyperplasia (left, scale bar, 2 μm) and in a higher magnification by the picture of a capillary loop with subendothelial electron dense deposits, basement membrane duplication and cellular interposition (right, scale bar, 1 μm). Asterisks indicate electron dense deposits. **D**, **F** Blood-Urea-Nitrogen (BUN) in serum of diseased (D), 12-week-old (E), and 24-week-old (F) mice. *n* ≥ 3 mice per group, early and late disease animals mixed. Data are presented as scatter dot plots with means indicated as a line. Statistical analysis was examined by one-way ANOVA with Tukey's multiple comparison post-test, *p* values are considered as follows: *p < 0.05, **p < 0.01, and ***p < 0.001 and indicated only for the diseased groups (BCL2^tg^ *vs*. E/R^tg^;BCL2^tg^).

We detected immune cell infiltrates in several organs, including lung, liver and kidney of BCL2^tg^ and E/R^tg^;BCL2^tg^ animals (Figure 2A, 2C, Figure 3A, Table S1, complete panels of lung and liver in Figure S3A, B), but not in E/R^tg^ or wildtype mice (Figure S4).

To test for the extent of dissemination the area of infiltration per organ was quantified. Interestingly, lungs, livers and kidneys of E/R^tg^;BCL2^tg^mice showed higher infiltration rates when compared to BCL2^tg^ mice (Figure 2B, 2D, Figure 3B). Some large infiltrates even exhibited follicular structures, reminiscent of follicular lymphoma as judged by two independent board-certified pathologists blinded to the underlying genotype. Infiltrates were composed of lymphocytes as well as plasma cells (most abundant in kidney infiltrates); we observed positive staining for CD79a and CD138, and to a lesser extent for CD3 (Figure 2E). However, we failed to detect any signs of chronic inflammatory processes such as vascular proliferation, nor did we detect severe signs of tissue injury, such as fibrosis. This argues for the lymphoma representing the source of infiltrating B cells. In summary, these data suggest that follicular B cell lymphoma formation in diseased E/R^tg^;BCL2^tg^ mice caused massive organ infiltration.

### BCL2^tg^ and double transgenic mice develop follicular lymphoma

Lymphoma formation was observed in double transgenic as well as BCL2^tg^ animals, in line with a previous report [36]. These lymphomas were composed mainly of centrocytes, some centroblasts and diffuse areas of plasma cell infiltration (Figure S5A). Furthermore, lymphomas of double transgenic and BCL2^tg^ mice displayed neoplastic capsular infiltration (Figure S5A). Tingible body macrophages were absent within the follicular structures in lymphomas and spleens (Figure S5B). Additionally, lymphomas were positive for only one light chain, namely Igλ (Figure S5C). This histopathological appearance is in line with the diagnosis of follicular lymphoma with plasmacytic differentiation in some of the cases (Figure S5D). Neoplastic follicular structures were enlarged as indicated by staining for the germinal center marker peanut agglutinin (PNA) which was - additionally to CD138^+^ cells - also detected in spleens of double transgenic and BCL2^tg^ mice (Figure S5E). In addition, all analyzed lymphomas exhibited a class-switched B cell phenotype. Immunohistochemical analysis of immunoglobulins on lymphoma infiltrates revealed positive staining for IgA and IgG, but not for IgM, in double transgenic and BCL2^tg^ mice (Figure S6A). Overall, lymphoma pathology was more pronounced in double transgenic mice.

### Glomerulonephritis in double transgenic mice is more pronounced than in BCL2^tg^ mice

Consistent with the macroscopic appearance of kidneys derived from diseased E/R^tg^;BCL2^tg^ and BCL2^tg^ animals (Figure 1H) we found pronounced pathological abnormalities histologically, immuno-histochemically and ultrastructurally (Figure 3A–3C). We detected changes consistent with highly active glomerulonephritis, tubular dystrophy and protein casts in addition to lymphoma infiltrates. Periodic acid Schiff (PAS) staining of kidney sections revealed marked glomerular mesangial matrix and cell increase. In addition, acid fuchsin orange G (AFOG) staining demonstrated glomerular protein deposits suggestive of immune complex deposition in both, double transgenic and BCL2^tg^ mice, but not in wildtype and E/R^tg^ mice (Figure 3A). E/R^tg^ mice developed glomerular pathology only with age, showing histologically mild mesangial cell and matrix increase which corresponded to mesangial and subendothelial electron dense deposits by transmission electron microscopy (Figure 3C). This phenotype was enhanced in BCL2^tg^ mice that showed endocapillary hypercellularity, mesangial matrix increase with mesangial and subendothelial Ig-containing immune deposits. Electron microscopy analysis (Figure 3C) confirmed a heterogeneous immune complex type glomerulonephritis, in line with a previous report [36], with prominent mesangial, subendothelial and occasional subepithelial electron dense deposits with basement membrane duplications and cellular interposition. Thus, the pattern of pathological changes is characteristic of a membranoproliferative pattern of injury similar to the type of glomerulonephritis observed in systemic diseases like lupus erythematosus. In double transgenic mice the mesangial and subendothelial electron dense deposits were even more pronounced. Glomeruli from these animals also exhibited segmental necrosis and formation of cellular crescents (Figure 3A, 3C). In general, signs of active glomerulonephritis were more pronounced in animals with early onset of disease (Figure 3A, AFOG staining) and, among all mice analyzed up to 30 weeks of age, the described kidney pathology was more severe in double transgenic compared to BCL2^tg^ mice. While only few diseased BCL2^tg^ mice older than 30 weeks presented with renal disease, all double transgenic mice older than 30 weeks suffered from severe glomerulonephritis.

Next, we addressed renal function by measuring blood urea nitrogen (BUN) in terminally diseased mice. BUN levels were significantly elevated in both, double transgenic as well as BCL2^tg^ mice when compared to age-matched wildtype and E/R^tg^ mice (Figure 3D). To test whether kidney damage starts earlier in double transgenic mice, we examined BUN levels in mice at 12 and 24 weeks of age. BUN was in a similar range in all tested mice at 12 weeks of age (Figure 3E). At 24 weeks of age we observed higher BUN levels in E/R^tg^ mice than in wildtype animals (Figure 3F). In line, double transgenic mice also showed higher BUN levels compared to BCL2^tg^ mice.

Taken together, our data indicate that glomerulonephritis is accelerated and more severe in kidneys of E/R^tg^;BCL2^tg^ than of BCL2^tg^ animals.

### Combined expression of ETV6/RUNX1 and BCL2 increases the development of immunoglobulin secreting plasma cells

**Figure 4:**
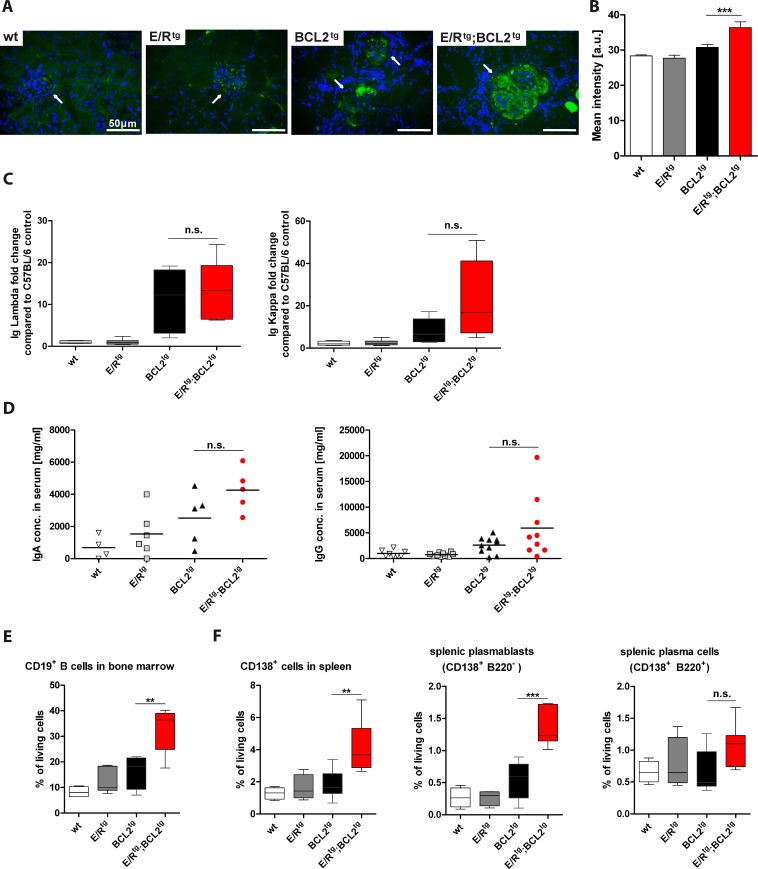
E/R^tg^;BCL2^tg^ mice have higher B cell numbers and immunoglobulin titers than BCL2^tg^ mice **A** Fluorescence immunohistochemistry showing representative IgG deposits (green) in glomeruli, nuclei counterstained with DAPI (blue). Glomeruli are indicated by arrows, scale bars, 50 μm. **B** Quantification of mean IgG intensity in glomeruli (*n* = 19 - 29 glomeruli per group) of at least three mice per group, including early and late disease mice in each group. Data are shown as means ± SEM. **C** ELISA of Ig Lambda (left panel) and Ig Kappa (right panel), normalized to a wt control. Relative fold change to the control mouse is indicated for Ig Lambda and Kappa, *n* = 4-7 mice per group, including both, early and late disease mice. **D** Quantification of serum IgA (*n* = 4-6 mice per group) and IgG (*n* = 8-10) levels. Data are presented as scatter dot plots with means indicated by a line. (**E**-**F**) Flow cytometric analysis of B cells (E), plasma cells and plasmablasts (F) is shown. (E) Percentage of CD19^+^ B of living cells in bone marrow are shown, *n* = 4-7 mice per group. (F) CD138^+^ cell, plasmablast- and plasma cell levels in spleen are indicated, *n* = 4-8 mice per group. (C, E, F) Data are visualized as box plots, whiskers indicating min and max. For all: Statistical analysis was performed using one-way ANOVA with Tukey's multiple comparison post-test, *p* values are considered as follows: *p < 0.05, **p < 0.01, and ***p < 0.001 and indicated only for the diseased groups (BCL2^tg^ *vs*. E/R^tg^;BCL2^tg^).

To test whether the increased protein deposits observed by AFOG staining are derived from immune complexes we analyzed IgG deposits in the kidneys. We detected significantly higher amounts of IgG in double transgenic mice as compared to all other groups (Figure 4A, 4B).

These data could indicate that ETV6/RUNX1 might impact on immunoglobulin production or autoimmune B cell generation. To test this hypothesis, we measured immunoglobulin levels in sera of terminally diseased mice. We observed elevated amounts of immunoglobulin kappa (Igκ) and lambda (Igλ) in diseased double transgenic mice compared to all other groups (Figure 4C). In addition, we found elevated IgA and IgG levels in sera of double transgenic mice when compared to all other cohorts (Figure 4D). In line, immunohistochemical analysis on plasma/B cell infiltrates in the kidney revealed higher numbers of IgA^+^ cells in double transgenic mice compared to BCL2^tg^ animals (Figure S6B).

Next we tested whether the increased levels of immunoglobulins in double transgenic mice were caused by a higher rate of Ig-producing plasma cells. Indeed, we observed a significantly increased percentage of CD19^+^ B cells in the bone marrow of double transgenic animals (Figure 4E). In the spleen, the percentage of CD138^+^ cells was significantly increased in double transgenic animals when compared to the other groups (Figure 4F). In particular, the frequency of CD138^+^B220^−^ plasmablasts was significantly elevated while the one of CD138^+^B220^+^ plasma cells was only slightly increased in double transgenic compared to healthy and BCL2^tg^ mice (Figure 4F). A similar trend was observed for absolute cell numbers of bone marrow CD19^+^ B cells, splenic CD138^+^ cells and plasmablasts (Figure S7A, B).

**Figure 5:**
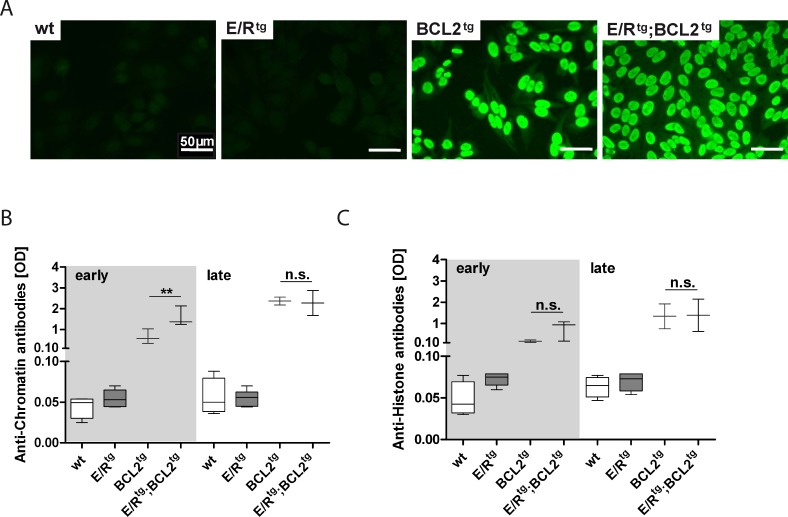
Double transgenic and BCL2^tg^ animals have elevated levels of autoantibodies in serum **A** Immunofluorescence pictures of ANA in BCL2^tg^ and double transgenic animals, scale bar, 50 μm. **B**, **C** ELISA of serum autoantibody levels, OD values of anti-chromatin (B) and anti-histone (C) are shown. *n* = 2-6 sera per group, whiskers indicate min and max. Data are presented as means ± SEM. Statistical analysis was performed using one-way ANOVA with Tukey's multiple comparison post-test, *p* values are considered as follows: *p < 0.05, **p < 0.01, and ***p < 0.001 and indicated only for the diseased groups (BCL2^tg^ *vs*. E/R^tg^;BCL2^tg^).

BCL2^tg^ mice were reported to develop a kidney disease with the histologic appearance of autoimmune glomerulonephritis [36]. Thus, we were interested whether autoantibodies are the driving force for glomerulonephritis progression in E/R^tg^;BCL2^tg^ animals. Significant amounts of anti-nuclear antibodies (ANA) could be detected in the serum of terminally sick double transgenic and BCL2^tg^ mice by immunofluorescence in HEp-2 cells (Figure 5A). In addition, an autoantibody profile (including anti-histone, anti-chromatin and antibodies directed against nuclear ribonucleoprotein - anti-nRNP, also known as anti-U1RNP) revealed increased levels of anti-histone and anti-chromatin antibodies in double transgenic and BCL2^tg^ mice, while we failed to detect any significant autoantibody titers in healthy wildtype or E/R^tg^ mice (Figure 5B, 5C). Within the early disease group showing enhanced glomerulonephritis, double transgenic sera exhibited higher levels of anti-chromatin and anti-histone antibodies. In the late phase sera from both double transgenic and BCL2^tg^ mice contained similarly elevated levels of autoantibodies. In addition, weak reactivity to the U1A protein of the U1RNP was observed in two of the six double transgenic sera tested whereas the sera of the other mouse groups were negative for antibodies to U1RNP and other autoantibodies analyzed (data not shown).

Taken together our data show a synergistic effect of combined ETV6/RUNX1 and BCL2 overexpression in development of B cell-dependent diseases. This was evident for both follicular lymphoma development, which led to significant organ infiltration in double transgenic mice, as well as for elevated autoimmune and plasmablast numbers. Interestingly, increased immunoglobulin levels in double transgenic mice resulted in more pronounced autoimmune glomerulonephritis when compared to BCL2^tg^ mice.

## DISCUSSION

Deregulated expression of BCL2 family members has been described in the context of ETV6/RUNX1 positive leukemia [18, 28, 29]. Here we provide evidence for cooperative effects of the antiapoptotic protein BCL2 with the chromosomal fusion product ETV6/RUNX1 *in vivo*. We intercrossed *ETV6/RUNX1* transgenic with *Vav-BCL2* transgenic mice. The characterization of this mouse model indicates that B-cell restricted expression of *ETV6/RUNX1* - resulting in elevated numbers of B cells - in concert with antiapoptotic *BCL2* expression - enhancing B cell survival - promotes increased immunoglobulin production, particularly autoimmune antibodies. In sum, these two hits resulted in the clinical picture of immune complex deposition in kidney glomeruli and in accelerated development of immune complex glomerulonephritis in E/R^tg^;BCL2^tg^ mice.

Overexpression of BCL2 is widely accepted as a hallmark of follicular lymphoma in humans. In mice, expression of BCL2 in the hematopoietic compartment has been described to result in glomerulonephritis with an incidence of 15 - 25% at 40 weeks and in the development of lymphomas with an incidence of 37 - 50% at 18 months of age [36]. While both disease phenotypes were aggravated in E/R^tg^;BCL2^tg^ animals compared to *Vav-BCL2* transgenic mice, we did not detect B-ALL development. Only one case of 16 analyzed double transgenic animals developed myeloid leukemia (Figure S2A-C). We speculate that this single case of leukemia is due to (an) additional mutation(s) on top of ETV6/RUNX1 and BCL2 expression and it would be of interest to define this in future analysis. The vast majority of mice, however, developed lymphoma and/or glomerulonephritis causing death before one year of age.

Certainly, it is of relevance at which stage of B cell development the transgene is expressed. *ETV6/RUNX1* expression in our model started at the level of committed B cells (CD19^+^ B cells). Thus, placing the *ETV6/RUNX1* transgene under control of the *Cd19* promoter may be too late to allow for B cell progenitor leukemia development when combined with BCL2 overexpression, but still allowing to promote autoimmune mature B cell abnormalities that aggravate glomerulonephritis.

Of note, overexpression of BCL2 also overcomes a critical barrier for the accumulation and expansion of B cell progenitors in the context of *Runx1* deficiency, - a situation also mimicked by the expression of the *ETV6/RUNX1* fusion gene in t(12;21) ALL [37]. In line, we observed the highest frequency of B cells in the double transgenic animals. Furthermore, another link between BCL2 and Runx1 has been demonstrated in T cells, where BCL2 has been described to protect from apoptosis induced by lack of Runx1 [38]. Interestingly, BCL2 was also shown to be essential for the survival effect of RUNX1 in human MLL fusion leukemia [39]. However, the detailed mechanistic understanding of this interaction is still missing.

In healthy individuals self-reactive B cells are eliminated through several mechanisms, including deletion, anergy and receptor editing [40–42]. To achieve immune tolerance, autoreactive lymphocytes must be negatively selected upon encounter with self-antigen. BCL2 has been reported to be highly expressed in pro-B cells, mature B lymphocytes and plasma cells, and downregulated at stages where negative selection occurs, such as the pre-B, immature B and germinal center (GC) B cell stages [43–45]. Constitutive overexpression of BCL2 in B cells has been shown to impair B cell tolerance induction in a number of models [46–49] protecting autoreactive B cells from deletion. Thereby, enhanced expression levels of BCL2 contribute to development of autoimmune diseases [24, 50, 51]. In line with our findings expression of BCL2 driven by the *Vav* promoter has been shown to be associated with development of heterogeneous autoimmune glomerulonephritis [36]. B cell-restricted expression of ETV6/RUNX1 in concert with *Vav* promoter-driven BCL2 expression further enhanced B cell autoreactivity in double transgenic mice. Thus, ETV6/RUNX1 expression contributes to increased B cell tolerance, which aggravates autoimmune disease.

A comparison of transgene expression levels between two other *BCL2* transgenic mouse lines (*Eμ-BCL2* 22 and *Eμ-BCL2* 36) and the *Vav-BCL2*^tg^ mice revealed that transgene expression does not strictly correlate with B cell numbers [36]. All three mouse lines show markedly elevated B cell levels, spontaneous production of antibodies against nuclear antigens and a large proportion of mice display immune-complex glomerulonephritis [24, 36]. Yet only pan-hematopoietic expression of BCL2 *via* the *Vav-BCL2* transgene led to follicular lymphoma development. Most likely this was due to the fact that only the *Vav-BCL2*^tg^ mice also have elevated T-lymphocyte levels and thus may have sufficient CD4^+^ T cell help to sustain efficient maturation of the excess B cells [36]. This suggests that not only BCL2 expression levels but also the cell lineage from which the transgene is expressed impact on the disease phenotype observed. However, as the ETV6/RUNX1 fusion is expressed from committed B cells onwards *via* the *Cd19* promoter, B cell levels in double transgenic mice are even more elevated than in BCL2^tg^ mice (Figure 4E, 4F and Figure S7A, B). In contrast to that the numbers of CD4^+^ and CD8^+^ T cells remained largely unaffected (Figure S7C, D). This might explain why the impact on glomerulonephritis is more pronounced than on follicular lymphoma in double transgenic mice.

The *AICDA* gene product, activation-induced cytidine deaminase (AID), is required for B cell tolerance in humans. As AID is the key molecule responsible for somatic hypermutation, class switch recombination and, in birds, gene conversion [52–54], it is interesting to note that very recently AID was also reported to drive - together with RAG1 and RAG2 - leukemic clonal evolution by contributing to the acquisition of genetic lesions in B-ALL [55]. Abundant *AICDA* and *RAG1* mRNA was correlated with poor outcome in ALL patients. The authors claimed that the physiological process of antibody diversification is twisted in ETV6/RUNX1 positive pre-leukemic B cell clones (with repeated exposure to inflammatory stimuli), which finally leads to overt disease. In addition, *AICDA* expression during B cell development was reported to be necessary for the production of anti-RNA IgG autoantibodies in a mouse model of systemic lupus erythematosus [56]. However, we did not observe significant changes in *Aicda* expression levels in double transgenic animals (Figure S8). We just noted increased class switch events in double transgenic animals associated with elevated levels of immunoglobulins (IgA, IgG) in sera of E/R^tg^;BCL2^tg^mice. The fact that impairment of renal function, as indicated by BUN levels, started between the age of 12 and 24 weeks strengthens our hypothesis of accumulation of antibody complexes. Moreover, we detected higher BUN levels in E/R^tg^ mice, which could be the result of elevated B cell numbers in lymphoid organs in these animals.

Interestingly, renal infiltration resulting in kidney enlargement was reported for up to 47% of children diagnosed with ALL [57–62]. Similarly, renal involvement in lymphoma is not uncommon, lymphoma infiltration into the kidney in rare cases even causes acute renal failure [63]. However, although a possible link between a genetic predisposition factor in the pathogenesis of autoimmunity and leukemogenesis has been recently proposed [64], we are not aware of any case reports for ETV6/RUNX1^+^ leukemia patients associated with autoimmune disease (and/or glomerulonephritis). Nevertheless, it is tempting to speculate that in follicular lymphoma patients (which typically display BCL2 overexpression) with strong kidney infiltration ETV6/RUNX1 target genes could be involved. In addition, as the *ETV6/RUNX1* fusion is frequently observed in newborn children, individual autoimmune or immune complex glomerulonephritis patients harboring the *ETV6/RUNX1* fusion (in plasma B cells) could exist exhibiting an aggravated glomerulonephritis phenotype. Our data would suggest a possible co-morbidity of ETV6/RUNX1 and that patients with autoimmune phenotype should be screened for the presence of the *ETV6/RUNX1* fusion.

## MATERIALS AND METHODS

### Animals

Generation of CD19^+^ B cell-specific *ETV6/RUNX1*-expressing mice (E/R^tg^) and mice expressing human *BCL2* in the hematopoietic system (*Vav-BCL2* expressing mice; BCL2^tg^) was described [16, 35]. We intercrossed these two mouse strains to generate double transgenic mice (E/R^tg^;BCL2^tg^) and maintained animals on a C57BL/6 background under specific-pathogen-free conditions. Mice that express only one (E/R^tg^ and BCL2^tg^, respectively) or no transgene (wildtype) were used as littermate controls. Daily monitoring for health status of mice and SPF housing in clean environment was ensured. Peripheral blood was first collected at 8 weeks of age and then every 6 weeks starting from 12 weeks of age. Mice were sacrificed and carefully analyzed at signs of disease (e.g., abdominal distension, ruffled fur, labored breathing, immobility, reduced food intake) according to ethical guidelines. All animal experiments were carried out according to an ethical animal license protocol approved by the Medical University of Vienna and the Austrian Federal Ministry of Science, Research and Economy.

### Blood smears and blood parameters

Blood smears were stained in a HEMA-TEK 2000 stainer with HEMA-TEK Modified Wright's Stain (Siemens Healthcare Diagnostics Inc., Tarrytown, NY). Confocal images were taken using an Axio Imager.Z1 microscope (Carl Zeiss, Oberkochen, Germany).

Quantification of blood parameters was obtained from EDTA blood by scil Vet ABC (Gurnee, IL) according to the manufacturers’ instructions.

### Histology and immunohistochemistry

Formalin-fixed, paraffin-embedded tissue sections were stained with hematoxylin-eosin (HE), periodic acid-Schiff (PAS) and acid fuchsin orange G (AFOG) according to standard procedures. Specific immunohistochemistry was performed on formalin-fixed, paraffin-embedded consecutive sections using the following antibodies: CD79a (24C2.5; eBioscience, San Diego, CA), CD3 (SP7; NeoMarkers, Freemont, CA), CD138 (281-2; BioLegend, San Diego, CA). Biotinylated peanut agglutinin [PNA] (Ref. B-1075; Vector Laboratories, Burlingame, CA) was used as a marker for germinal center cells. Characterization of infiltrates was performed using polyclonal antibodies against Kappa light chain (Ref. NB7549), Lambda light chain (Ref. NB7552), IgM (Ref. NB7497) and IgA (Ref. NB7504), all Novus Biologicals (Littleton, CO) as well as an anti-polyvalent biotinylated antibody against IgG (Ref. KIT-IDST1007; Empire Genomics, Buffalo, NY). To detect renal IgG deposits, an F(ab’)2 anti-mouse IgG-fluorescein isothiocyanate (FITC) antibody (eBioscience) was used as previously described [65].

### Quantitative immunohistochemistry

Quantification was performed using ImageJ software [66]. In more detail, glomeruli of at least three different mice per group were selected on 40x magnification pictures of kidneys and analyzed for staining intensities. For histological quantification of infiltrated areas, at least two consecutive 2.5 μm sections per mouse of at least three mice per group were stained and scanned with TissueFAXS^TM^ software (TissueGnostics GmbH, Vienna, Austria; http://www.tissuegnostics.com). Analysis was performed using HistoQuest^TM^ software (TissueGnostics GmbH, for details see [67]) as infiltrate area per organ area.

### Electron microscopy

For electron microscopy, 1-2 mm thin pieces of kidney were fixed in 4% paraformaldehyde resolved in 0.1 M cacodylate buffer and embedded in epoxy resin. Ultrathin sections were cut with an Ultracut EM UC7 (Leica Biosystems, Wetzlar, Germany), stained with uranylacetate and examined using a Jeol JEM 1010 (Jeol, Tokyo, Japan) electron microscope.

### Flow cytometric analysis

After incubation of single-cell suspensions with an anti-CD16/CD32 (93) antibody to prevent nonspecific Fc receptor-mediated binding, cells were stained with antibodies directed against murine Ly6G/Gr-1 (1A8-Ly6G), CD11b (M1/70), CD3e (145-2C11), B220 (RA3-6B2), CD19 (1D3), CD138 (281-2), CD4 (GK1.5) or CD8 (53-67). Fluorescein isothiocyanate (FITC)- phycoerythrin (PE)- or allophycocyanin (APC)-labeled Streptavidin was used for the detection of biotinylated antibodies. All antibodies were purchased from eBioscience or BD Biosciences (San Jose, CA), respectively. Subsequently, samples were acquired on a BD FACSCanto II flow cytometry device (BD Biosciences), and data were analyzed with FACSDiva (BD Biosciences) or FlowJo software (TreeStar, Ashland, OR).

### Serum biochemistry

Serum levels of urea were measured using Reflotron-based test strips (Roche Applied Science, Penzberg, Germany). Blood urea nitrogen was calculated using the following formula: Urea (mg/dl) x 0.467 = BUN (mg/dl).

### Quantification of immunoglobulin levels and autoantibody testing

An ELISA-based immunoglobulin (Ig) clonotyping system (Southern Biotech, Birmingham, AL) was used to determine Ig titers in the serum of mice. For quantification of IgA and IgG levels in serum the Mouse IgA and IgG ELISA Ready-SET-Go! kits (eBioscience) were utilized according to the manufacturer's protocol.

Anti-nuclear antibodies (ANA) were detected using VIRGO^®^ ANA/HEp-2 IgG cells (Hemagen Diagnostics, Columbia, MD) as a substrate coated on microscope slides. Mouse serum was added to the cells and ANA were visualized by addition of a FITC-labeled polyclonal rabbit anti-mouse immunoglobulin antibody (Ref. F 0261; Dako, Glostrup, Denmark) as described [63].

Anti-histone and anti-chromatin (nucleosome) antibodies were measured by ELISA (Inova Diagnostics, San Diego, CA) using horseradish peroxidase-conjugated anti-mouse antibodies (Dako; 1:2000) as secondary antibody as previously described [68].

INNO-LIA^®^ ANA Update immunoassay (Fujirebio, Malvern, PA) was used for the detection and identification of autoantibodies against nuclear and cytoplasmic antigens in serum according to the manufacturer's protocols.

### Statistics

Statistical analyses were performed using GraphPad Prism 5 (San Diego, CA). Differences were investigated for statistical significance by one-way analysis of variance with Tukey's Multiple Comparison Test if not otherwise mentioned. For Kaplan-Meier plots significant differences were calculated by Log-rank test. Error bars represent means ± SEM if not stated otherwise. *p* values are considered as follows: *p* < 0.05 *, *p* < 0.01 **, and *p* < 0.001 ***.

## SUPPLEMENTARY MATERIAL FIGURES AND TABLE


