# Supplementary material for: Cooperation of ETV6/RUNX1 and BCL2 enhances immunoglobulin production and accelerates glomerulonephritis in transgenic mice
Source: Oncotarget. 2016 Feb 23;7(11):12191–205. doi: 10.18632/oncotarget.7687 (PMC4914278; doi:10.18632/oncotarget.7687)
Supplement: Supplementary file 1 [file oncotarget-07-12191-s001.pdf]

Cooperation of ETV6/RUNX1 and BCL2 enhances immunoglobulin production and accelerates glomerulonephritis in transgenic mice

Supplementary Material

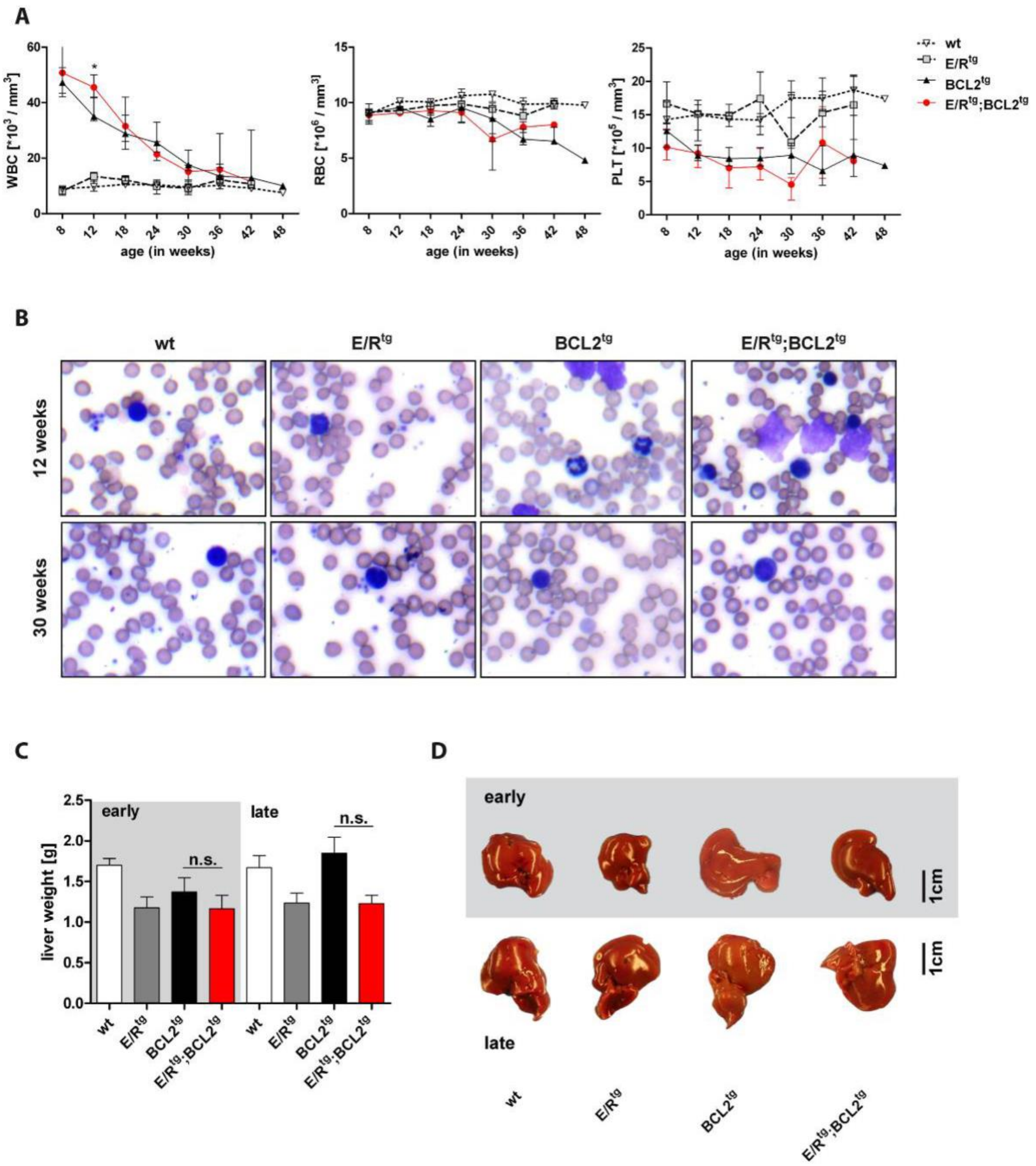

Supplementary Figure S1: E/R^{tg};BCL2^{tg} and BCL2^{tg} mice show enhanced white blood cell levels at young age that decrease with progressing disease.
(**A**) White and red blood cell levels and platelets as measured over time. Plots show median and error, as well as interquartile range, initially n ≥ 9 mice per time point and group. (**B**) Representative blood smears at 12 and 30 weeks of age. (**C**) Statistical analysis of liver weight, n ≥ 3 mice per group, means ± SEM are shown. (**D**) Representative pictures of livers of early and late disease mice and their corresponding controls. Scale bar, 1 cm. For all: Statistical analysis was performed using one-way ANOVA with Tukey's multiple

comparison post-test, p values are considered as follows: * $p<0.05$, ** $p<0.01$, and *** $p<0.001$ and indicated only for the diseased groups ($BCL2^{tg}$ vs. E/R^{tg};$BCL2^{tg}$)

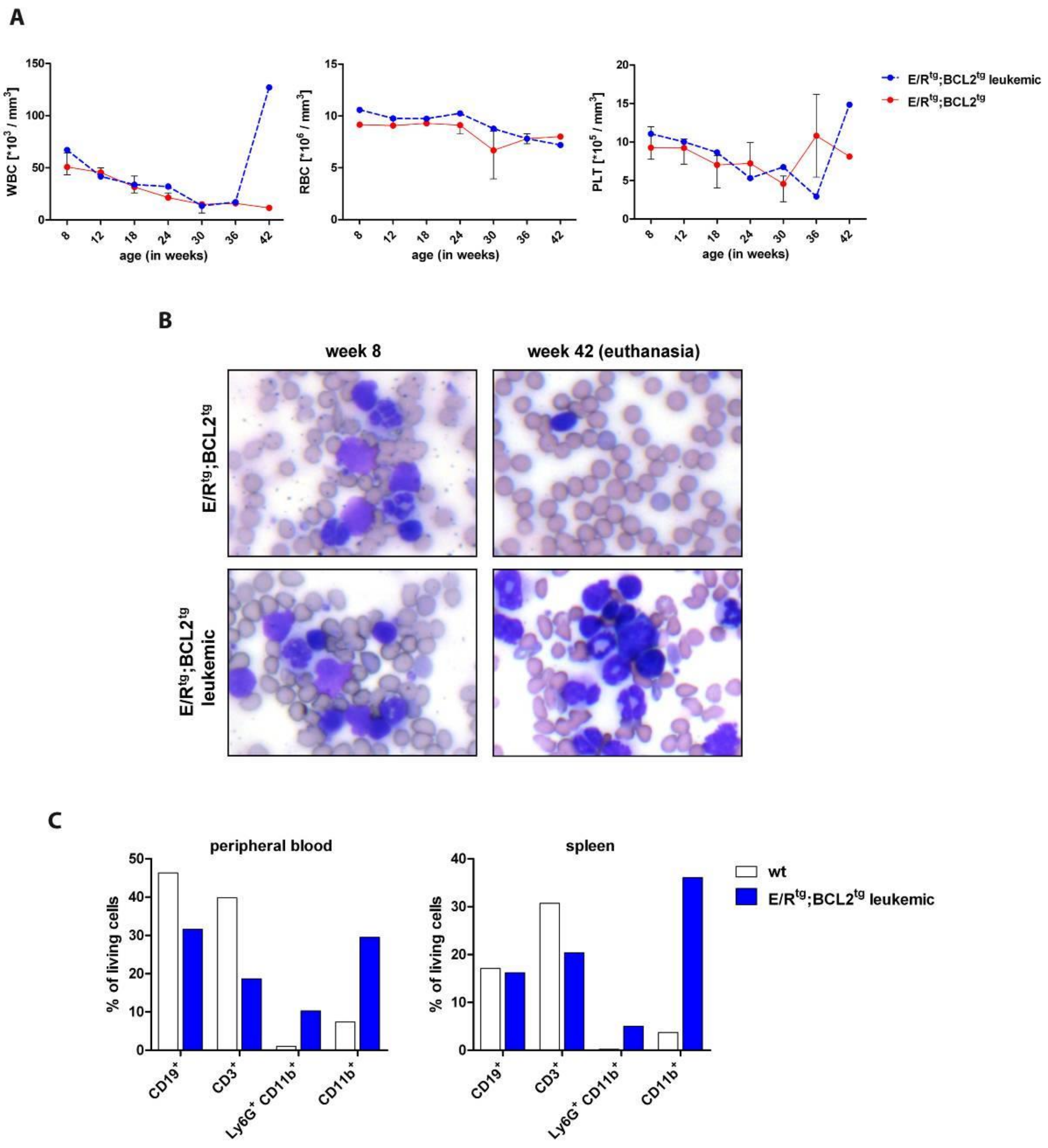

Supplementary Figure S2: Leukemia formation was observed in a single case of a double transgenic mouse.

(**A**) White and red blood cell levels and platelets of the leukemic mouse versus non-leukemic double transgenic animals as measured over time. Plots show median and error, as well as interquartile range, initially $n \geq 9$ mice per time point in the double transgenic group. (**B**) Blood smears of the leukemic mouse and a representative double transgenic control mouse at 8 weeks of age and at euthanasia (42 weeks). (**C**) B ($CD19^+$), T ($CD3^+$) and myeloid cells ($Ly6G^+$ $CD11b^+$ for monocytes, neutrophils and eosinophils, and $CD11b^+$ for monocytes) in peripheral blood and spleen of a wildtype (wt) and the leukemic mouse. Data are shown as bar graphs.

A

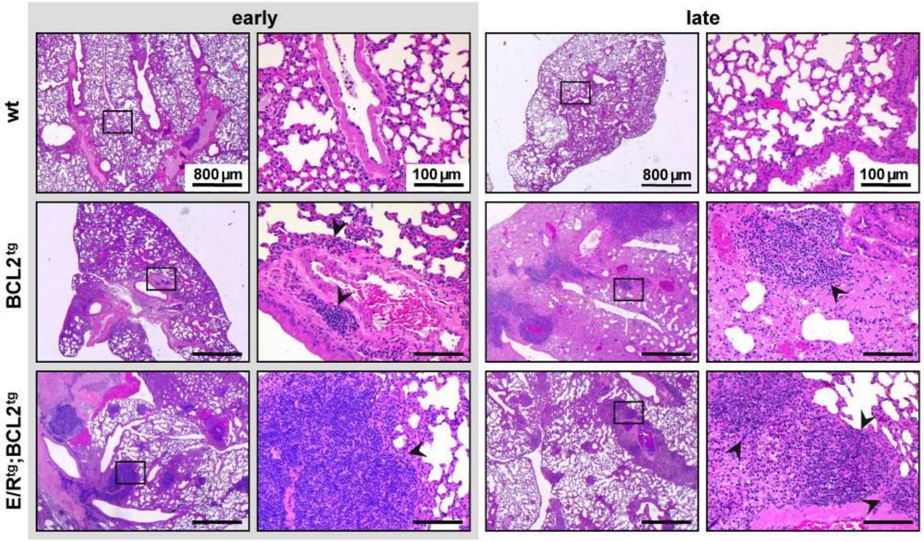

B

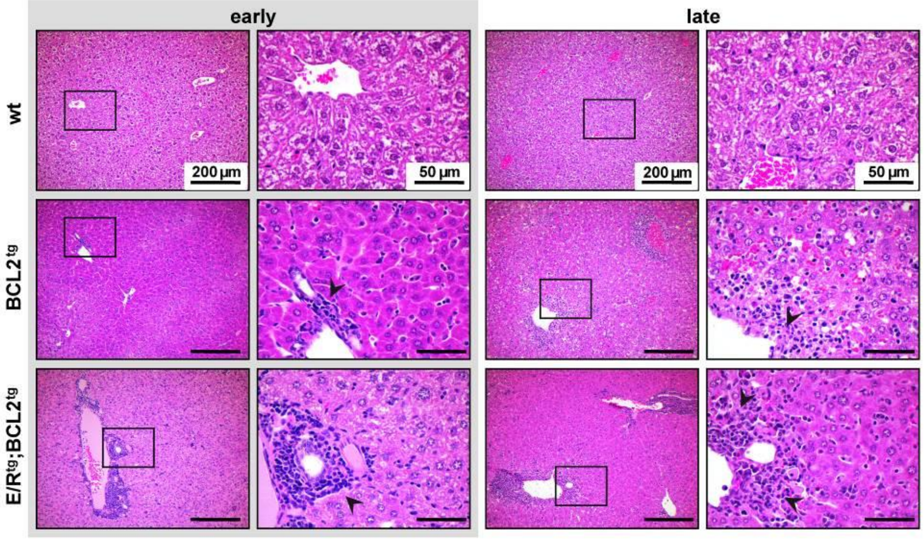

Supplementary Figure S3: Lungs and livers of BCL2^{tg} and E/R^{tg};BCL2^{tg} mice harbor lymphocyte infiltrates.

Representative HE stainings of lungs (**A**) and livers (**B**) of wt, BCL2^{tg} and double transgenic animals are shown. Infiltrations (arrowheads) are found alongside vessels. Scale bars are as indicated in the picture.

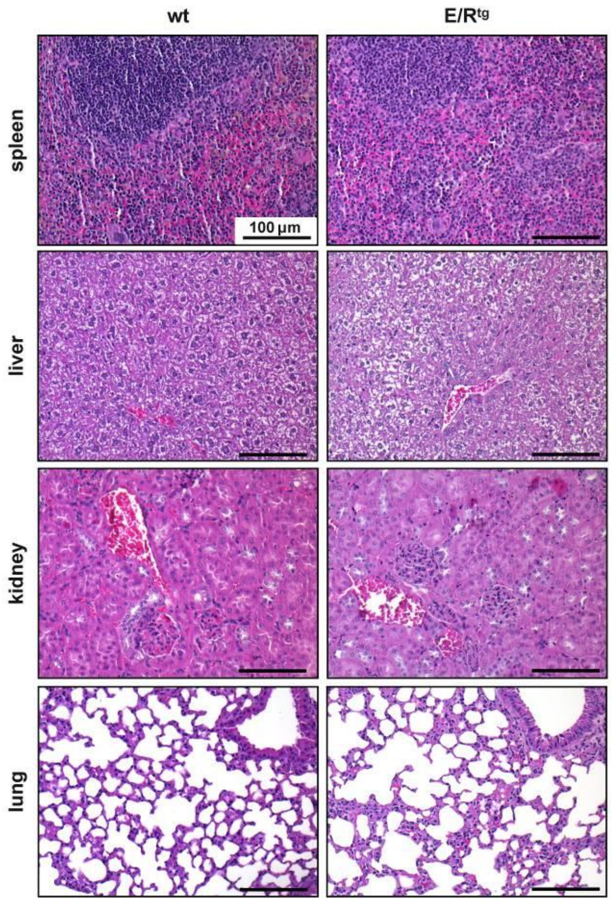

Supplementary Figure S4: Histological comparison of wt and E/R^{tg} mice.

Representative pictures of HE stained sections of spleen, liver, kidney and lung of wt and E/R^{tg} mice are shown. Scale bar, 100 µm.

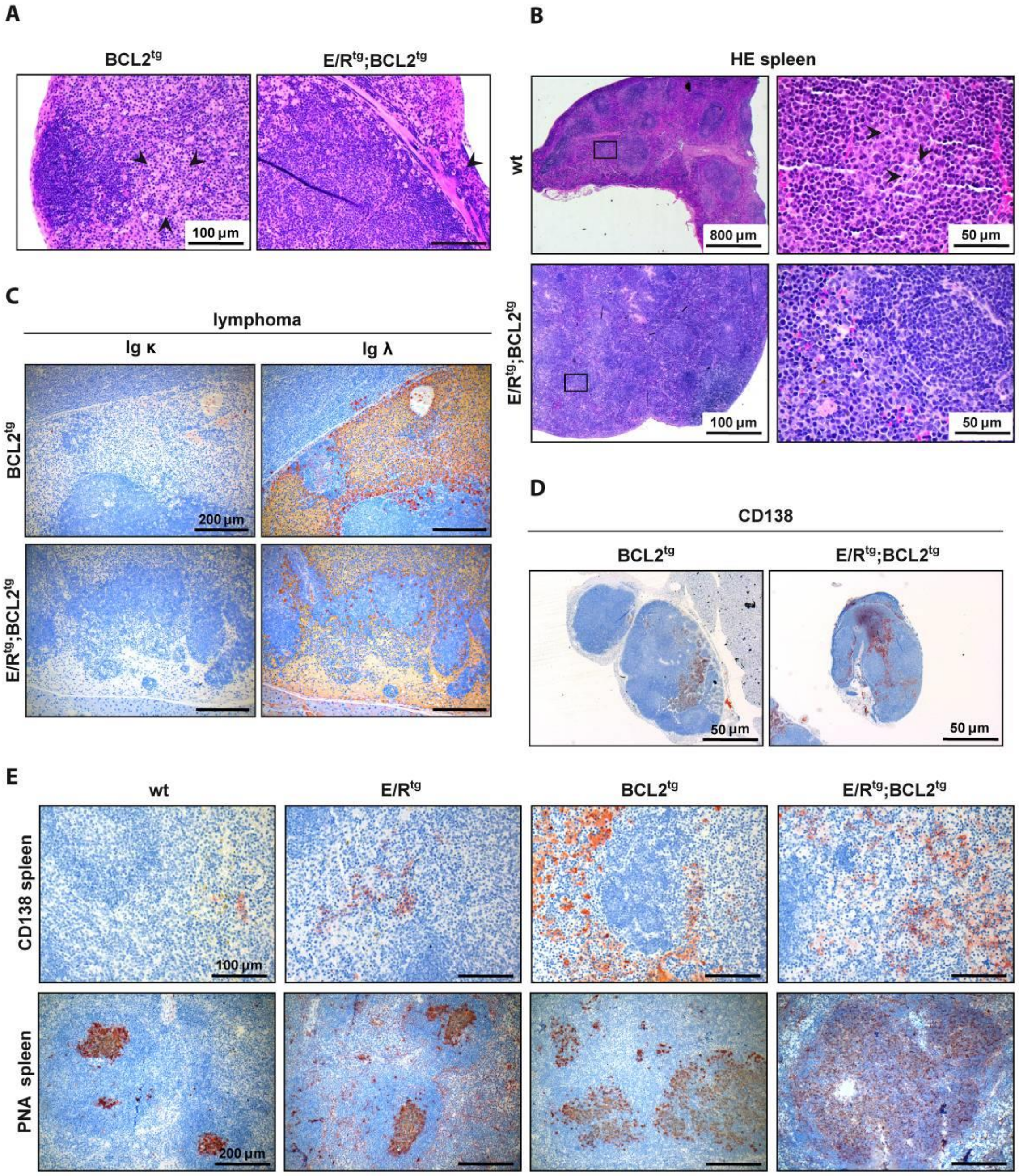

Supplementary Figure S5: BCL2^{tg} and double transgenic animals exhibit germinal center hyperplasia and lymphomas as well as disrupted splenic architecture.
(**A**) Plasma cells and centrocytes in lymph nodes of diseased BCL2^{tg} and E/R^{tg};BCL2^{tg} mice are exemplified by the picture of an HE stained lymph node derived from a BCL2^{tg} mouse (left; within arrowheads). Neoplastic growth beyond the capsule is illustrated by an HE stained section of a lymph node derived from an E/R^{tg};BCL2^{tg} mouse (right; arrowhead). One representative example is shown for each. Scale bars, 100 µm. (**B**) Representative spleen sections of wildtype and double transgenic mice. Spleens of double transgenic animals show cellular monotony at high power, indicated by uniform staining of germinal centers. Arrowheads in wt indicate tingible body macrophages, which are absent in double transgenic mice. Scale bars as indicated in the pictures. (**C**) Representative staining for Igκ and Igλ light chains on lymphoma sections of BCL2^{tg} and double transgenic mice, respectively. Scale bar, 200 µm. (**D**) Overviews of lymphoma sections of BCL2^{tg} and double transgenic animals stained for the plasma cell

marker CD138. Scale bar, 50 µm. (**E**) Sections derived from spleens of all mouse groups are depicted. Spleens of BCL2^{tg} and double transgenic mice show CD138^{+} plasma cells (upper panels) and enlarged neoplastic germinal centers indicated by peanut agglutinin (PNA) staining (lower panels). Scale bars as indicated in the pictures.

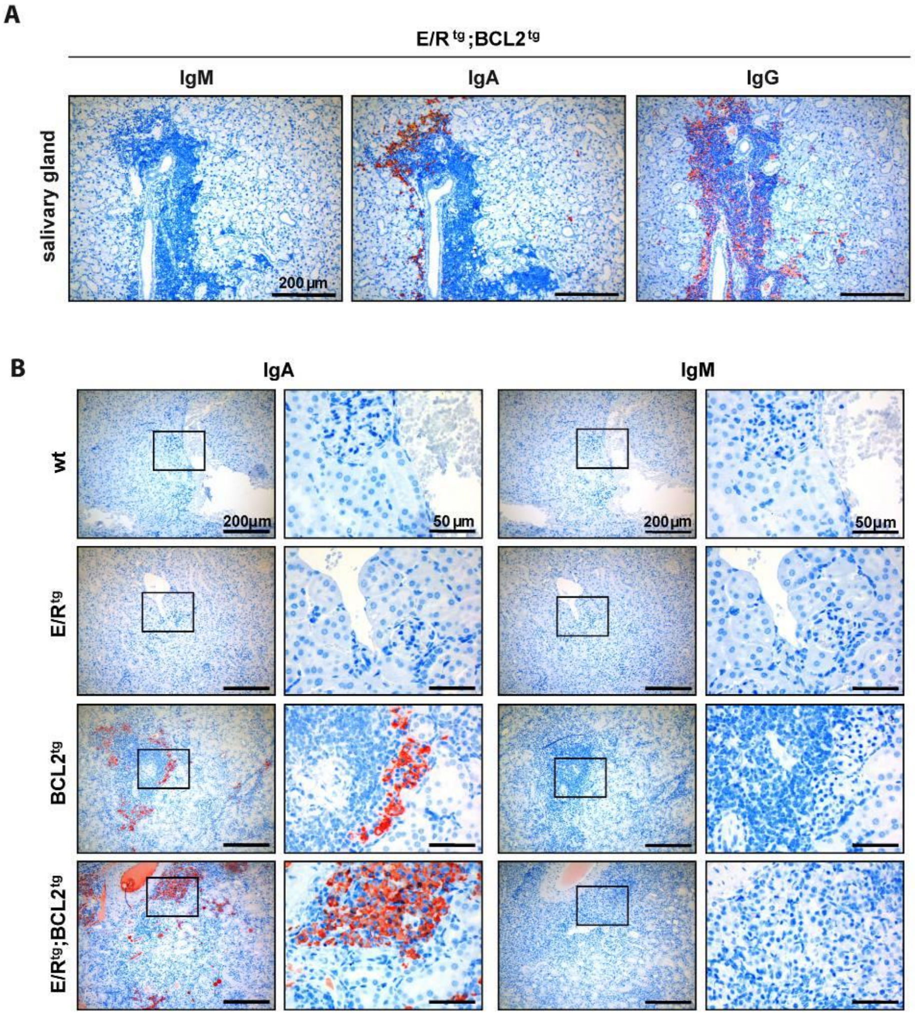

Supplementary Figure S6: Infiltrates indicate class-switched lymphoma cells.
(**A**) Representative staining for IgM, IgA and IgG on an infiltrate-containing salivary gland section of a double transgenic mouse. Scale bars, 200 µm. (**B**) Immunohistochemistry of IgA and IgM on kidney sections of all mouse groups. Scale bars as indicated in the pictures.

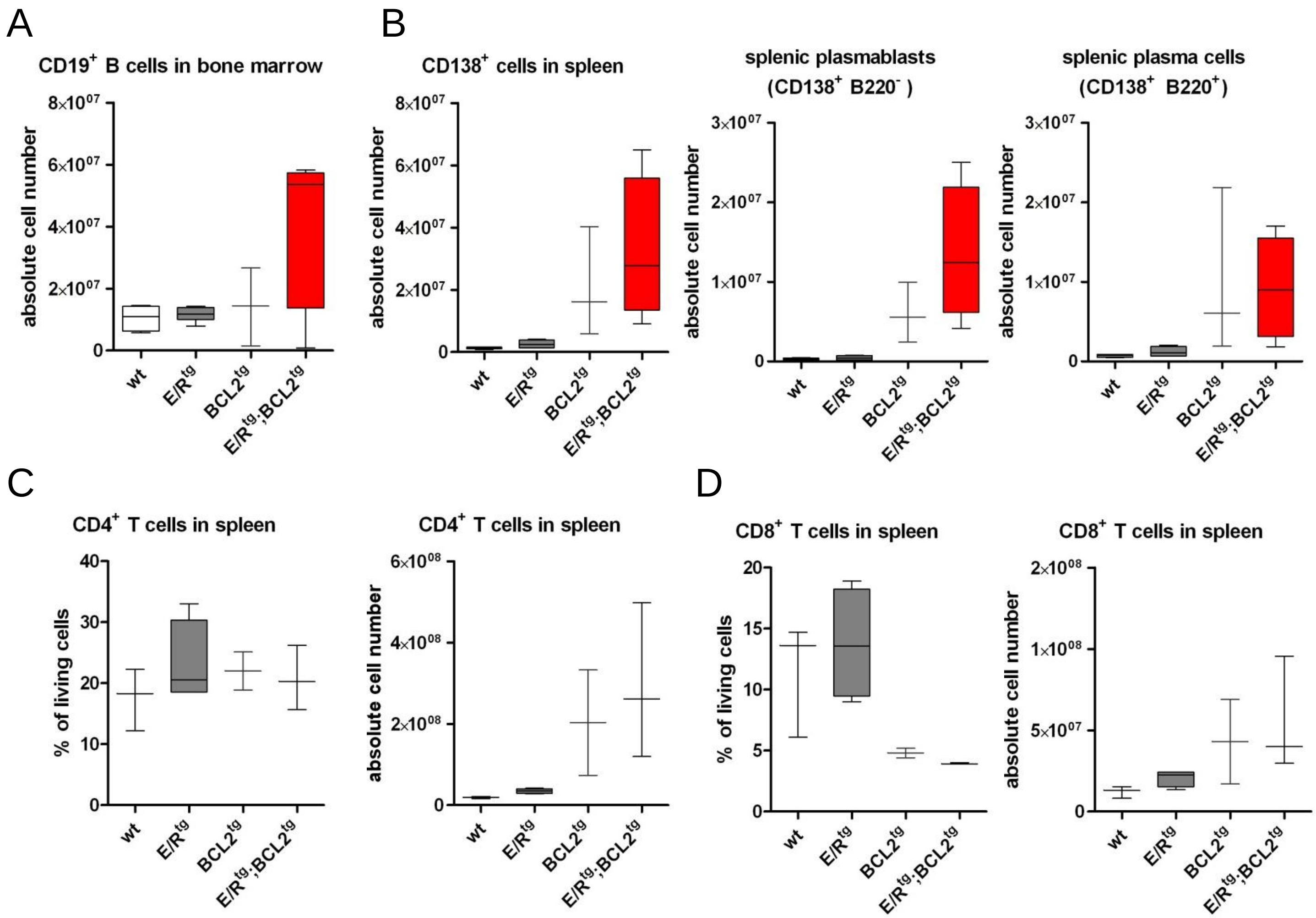

Supplementary Figure S7: E/R^{tg};BCL2^{tg} mice have higher B cell numbers while the numbers of T cells remain unaffected.

(**A**-**B**) Flow cytometric analysis of B cells (A), plasma cells and plasmablasts (B) is shown. (A) Absolute detected cell numbers of CD19^{+} B cells in bone marrow are shown, n = 3-4 mice per group. (B) CD138^{+} cell, plasmablast- and plasma cell levels in spleen are indicated via absolute cell numbers, n = 3-4 mice per group.

(**C**-**D**) Flow cytometric analysis of CD4^{+} (C) and CD8^{+} T cells (D) in spleen is shown. (C) Population of CD4^{+} T cells is shown as percentages of living cells and in absolute cell numbers in spleen, n = 2-4 mice per group. (D) Population of CD8^{+} T cells is shown as percentages of living cells and in absolute cell numbers in spleen, n = 2-4 mice per group.

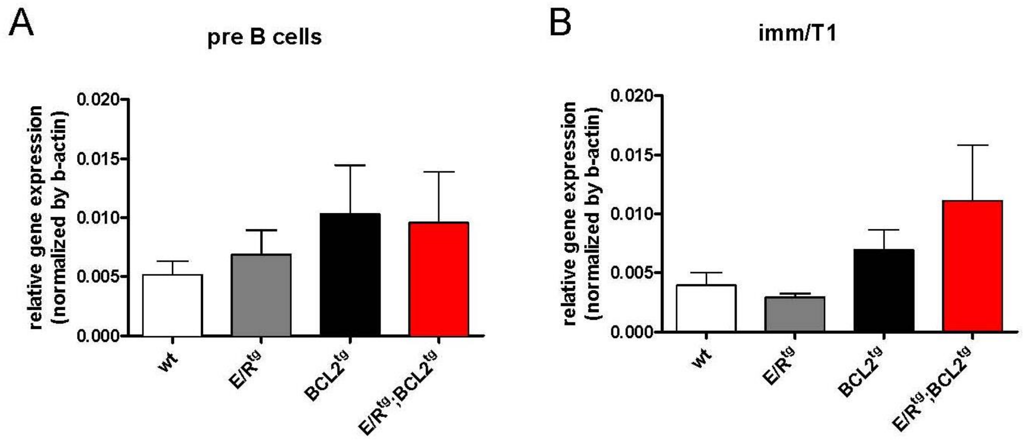

Supplementary Figure S8: *Aicda* gene expression in bone marrow B cell subpopulations.
(**A**-**B**) RT-qPCR analysis for *Aicda* of sorted bone marrow B cell subpopulation, namely pre B cells (A) and immature/T1 cells (B), is shown. (A) *Aicda* mRNA expression in sorted bone marrow-derived pre B cells of the four different mouse cohorts, n = 3 mice per group. (B) *Aicda* mRNA expression in sorted bone marrow-derived immature/T1 cells of the four different mouse cohorts, n = 3 mice per group

TABLES

Supplementary Table S1. Infiltrations in non-hematopoietic organs

Organ	$BCL2^{tg}$		$E/R^{tg};BCL2^{tg}$	
	Early disease	Late disease	Early disease	Late disease
**Liver**	**4/4 (100%)**	**5/5 (100%)**	**4/4 (100%)**	**3/3 (100%)**
**Lung**	**4/4 (100%)**	**4/5 (80%)**	**4/4 (100%)**	**3/3 (100%)**
**Kidney**	**4/4 (100%)**	**5/5 (100%)**	**4/4 (100%)**	**3/3 (100%)**
**Salivary gland**	**4/4 (100%)**	**4/4 (100%)**	**3/3 (100%)**	**3/3 (100%)**
**Heart**	0/4 (0%)	0/4 (0%)	0/3 (0%)	0/3 (0%)
**Brain**	0/4 (0%)	0/3 (0%)	0/4 (0%)	0/4 (0%)

Supplementary Table S1: The frequency of occurrence of infiltrates in various tissues of $BCL2^{tg}$ and double transgenic mice is summarized.

METHODS

FACS sorting, RNA isolation and RT-qPCR

Bone marrows (wt, $BCL2^{tg}$, E/R^{tg}, $E/R^{tg};BCL2^{tg}$) were isolated and cells were stained for 30 min at 4°C using antibodies (1:100) directed against CD45.1 PeCy5 (Clone A20, eBioscience, San Diego, USA), CD93 APC (Clone AA4.1, eBioscience), IgM FITC (R6-60.2, BD), IgD (Clone: 11-26c, eBioscience), Streptavidin APC eFluor780 (eBioscience), CD43 PE (Clone S7, BD). High-purity FACS-sorting was

performed at 4°C on a FACS Aria III device equipped with a 488 nm, 561 nm, 633 nm and 395 nm laser. Analysis was done using FACSDiva software (Becton-Dickinson, Vienna, Austria). $CD93^{+}/B220^{low}/IgM^{+}/IgD^{-}$ immature/T1 cells and $CD93^{+}/B220^{low}/IgM^{-}/IgD^{-}/CD43^{-}$ pre B cells were sorted directly into the lysis buffer of the RNeasy Micro Kit (Qiagen, Hilden, Germany) and RNA was isolated according to manufactorers protocol. Reverse transcription was performed using the RevertAid H Minus First Strand cDNA Synthesis Kit (Thermo Scientific, Waltham, USA) and real-time PCR analyses on the Mastercycler® ep realplex (Eppendorf, Hamburg, Germany) for *β actin*, and *Aicda* were executed with SYBR® Green I nucleic acid gel stain (life technologies, Carlsbad, USA), Taq DNA Polymerase (5 prime, Hilden, Germany) and the following primer sequences: *β actin* [foward GCTCATAGCTCTTCTCCAGGG, reverse CCTGAACCCTAAGGCCAACCG], and *Aicda* [forward AACCCAATTTTCAGATCGCG, reverse AGCGGTTCCTGGCTATGATAAC].
